# The Effect of Bergenin on Isonicotinic Acid Hydrazide and Rifampicin-Induced Liver Injury Revealed by RNA Sequencing

**DOI:** 10.3390/molecules28145496

**Published:** 2023-07-19

**Authors:** Ting Li, Chaoyue Yang, Houkang Cao, Siyi Mo, Bo Li, Zhipeng Huang, Ruobing Zhang, Jianzhao Wu, Kefeng Zhang, Ya Gao

**Affiliations:** 1 Pharmacology Laboratory of Prevention and Treatment of High Incidence of Disease, Guilin Medical University, Guilin 541199, China; liting1999@163.com (T.L.); loxxvwyr7532251@163.com (C.Y.); zyxchkchk@163.com (H.C.); siyimmm@163.com (S.M.); gszylibo@163.com (B.L.); 15077309262@163.com (Z.H.); zhangmengyuan7506@163.com (R.Z.); tjkerrywu@163.com (J.W.); 2 Guangxi Key Laboratory of Diabetic Systems Medicine, Guilin Medical University, Guilin 541199, China

**Keywords:** bergenin, isonicotinic acid hydrazide, rifampicin, liver injury, RNA sequencing

## Abstract

Bergenin (BER), a natural component of polyphenols, has a variety of pharmacological activities, especially in improving drug metabolism, reducing cholestasis, anti-oxidative stress and inhibiting inflammatory responses. The aim of this study was to investigate the effects of BER on liver injury induced by isonicotinic acid hydrazide (INH) and rifampicin (RIF) in mice. The mice model of liver injury was established with INH (100 mg/kg)+RIF (100 mg/kg), and then different doses of BER were used to intervene. The pathological morphology and biochemical indicators of mice were detected. Meanwhile, RNA sequencing was performed to screen the differentially expressed genes and signaling pathways. Finally, critical differentially expressed genes were verified by qRT-PCR and Western blot. RNA sequencing results showed that 707 genes were significantly changed in the INH+RIF group compared with the Control group, and 496 genes were significantly changed after the BER intervention. These differentially expressed genes were mainly enriched in the drug metabolism, bile acid metabolism, Nrf2 pathway and TLR4 pathway. The validation results of qRT-PCR and Western blot were consistent with the RNA sequencing. Therefore, BER alleviated INH+RIF-induced liver injury in mice. The mechanism of BER improving INH+RIF-induced liver injury was related to regulating drug metabolism enzymes, bile acid metabolism, Nrf2 pathway and TLR4 pathway.

## 1. Introduction

Isonicotinic acid hydrazide (INH) and rifampicin (RIF) are clinical first-line drugs for tuberculosis treatment [1], and they are the main drugs that cause drug-induced liver injury (DILI). The incidence of DILI ranges from 2.0% to 28% in the world [2]. DILI is a major cause of acute hepatitis, which leads to liver fibrosis and eventually liver cancer [3]. Under the action of N-acetyltransferase and amide hydrolase, INH metabolizes into hydrazine and acetyl hydrazine. Hydrazine has a direct toxic effect on hepatocytes. Concurrently, acetyl hydrazine is oxidized in the presence of CytochromeP450 (Cyp450) to produce reactive oxygen species (ROS) and other active intermediate products [4,5]. The combination of INH and RIF is more hepatotoxic than INH or RIF alone. The RIF is deacetylated, which provides acetyl for the acetylation of INH and accelerates the metabolism of INH. In addition, RIF can also increase drug-metabolizing enzyme activities and the metabolically active intermediate of acetyl hydrazine, which results in liver cell necrosis or apoptosis [6]. Although INH and RIF are so harmful to the liver, their use in patients is inevitable clinically. Consequently, it is of great significance to find effective drugs to alleviate INH+RIF-induced liver injury.

Although it has been shown that some drugs (such as Naringenin, Phloridzin) have a therapeutic effect on INH+RIF-induced liver injury [7,8], clinically, the treatment of liver injury caused by INH+RIF is still inconclusive. Many compounds in natural plants have been found to be effective in treating various diseases [9,10]. Bergenin (BER), a natural component of polyphenols, has good antitussive, expectorant, anti-inflammatory, antiviral and neuroprotective effects. Nowadays, BER is mainly used in the clinical treatment of chronic bronchitis [11]. BER has been shown to improve acute liver injury and liver fibrosis [12,13]. It has been reported that BER inhibited nuclear factor kappa B (NF-κB), and thus down-regulated tumor necrosis factor-α (TNF-α), interleukin-1β (IL-1β), myeloperoxidase (MPO) and cyclooxygenase-2 (COX-2) to attenuate hepatocyte death [14]. Our previous studies have also shown that BER improves oxidative stress caused by liver injury. This is due to the regulation of the p-JNK/JNK and P-AMPK/AMPK signaling pathways [15]. These studies suggest that BER plays a role in protecting the liver by improving oxidative stress and the inflammatory response. It has been shown that oxidative stress, inflammatory reactions and cholestasis are important inducements of liver injury [16]. However, whether BER can improve INH+RIF-induced liver injury and its mechanism remain unclear.

RNA sequencing is the transcriptome with a low background noise and no upper quantitative limit. It is characterized by a high degree of technical and biological repeatability. Furthermore, by comparing the gene sequences of all the transcriptional products with the known gene pool, RNA sequencing can find out the differentially expressed genes and the related signaling pathways. RNA sequencing plays an important role in revealing the potential targets of diseases and clarifying the mechanism of drug action [17]. Therefore, RNA sequencing was used to investigate the mechanism of BER alleviating INH+RIF-induced liver injury.

## 2. Results

### 2.1. Effects of BER on Liver Function

**Figure 1:**
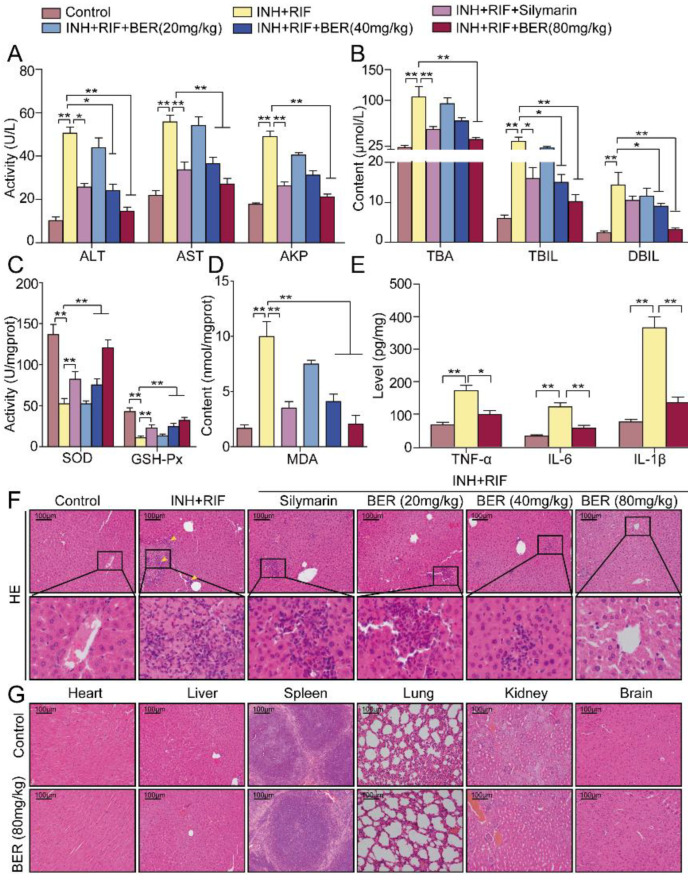
Effects of BER on liver function. (**A**) ALT, AST and AKP activities in serum. (**B**) TBA, TBIL and DBIL contents in serum. (**C**) SOD and GSH-Px activities in liver tissue. (**D**) MDA content in liver tissue. (**E**) TNF-α, IL-6 and IL-1β contents in liver tissue. (**F**) HE staining. (**G**) HE staining of the heart, liver, spleen, lung, kidney and brain in the Control group and BER (80 mg/kg) group. All data were presented as the means ± SEM (n = 10; * *p* < 0.05, ** *p* < 0.01). Bar = 100 μm, yellow arrow: focal necrosis and inflammatory cell infiltration.

Compared with the Control group, the activities or contents of ALT, AST, AKP, TBA, DBIL and TBIL in the INH+RIF group were significantly up-regulated. Compared with the INH+RIF group, they were significantly down-regulated after the BER intervention (Figure 1A,B).

Compared with the Control group, the activities of SOD and GSH-Px were significantly down-regulated, while the contents of MDA were significantly up-regulated in the INH+RIF group. Compared with the INH+RIF group, the activities of SOD and GSH-Px in the liver were significantly up-regulated, and the contents of MDA were significantly down-regulated after BER treatment (Figure 1C,D). Meanwhile, TNF-α, IL-6 and IL-1β levels were significantly up-regulated in the INH+RIF group. Compared with the INH+RIF group, they were significantly down-regulated after the BER intervention (Figure 1E).

HE staining showed normal hepatocyte structure without necrosis or inflammation in the Control group. However, massive focal necrosis and inflammatory cell infiltration was shown in the INH+RIF group (Figure 1F). After the BER intervention, the above symptoms were improved. Furthermore, the HE staining results of the BER (80 mg/kg) group showed no significant pathological differences in heart, liver, spleen, lung, kidney and brain tissues compared with the Control group (Figure 1G). This indicated that BER (80 mg/kg) had no significant toxic effects.

### 2.2. RNA Sequencing Analysis Results

#### 2.2.1. Overview of RNA Sequencing Analysis

**Figure 2:**
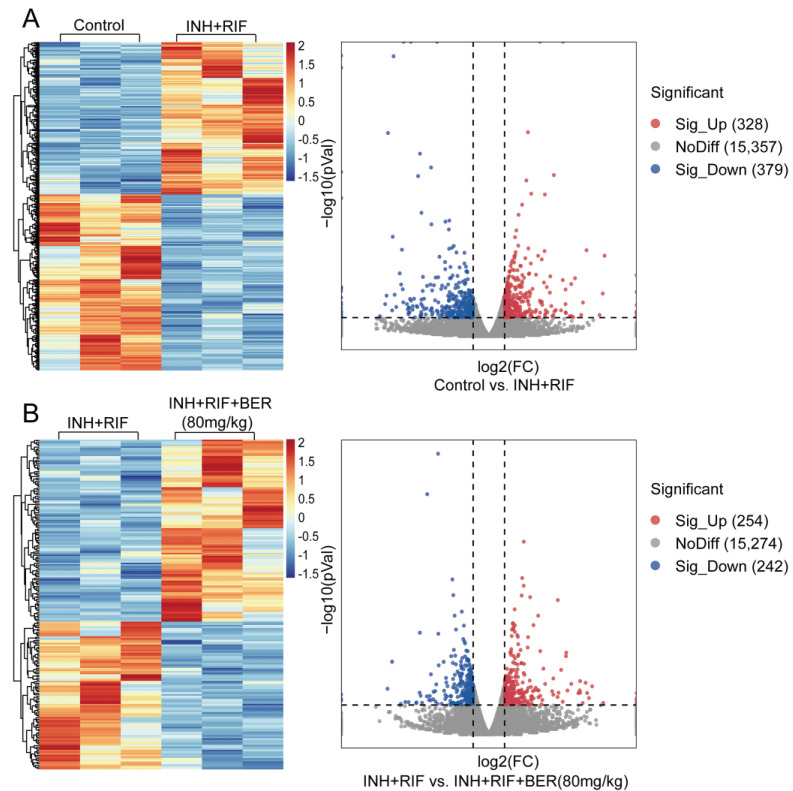
The hierarchical cluster analysis and scatter plot of all differentially expressed genes. (**A**) The Control group vs. the INH+RIF group. (**B**) The INH+RIF group vs. the INH+RIF+BER (80 mg/kg) group.

Compared with the Control group, 707 genes with significant changes were screened in the INH+RIF group, of which 328 were up-regulated and 379 were down-regulated (Figure 2A). Compared with the INH+RIF group, 496 genes were significantly changed after the BER intervention, of which 254 were up-regulated and 242 were down-regulated (Figure 2B).

#### 2.2.2. Effects of BER on Drug Metabolism Genes

**Figure 3:**
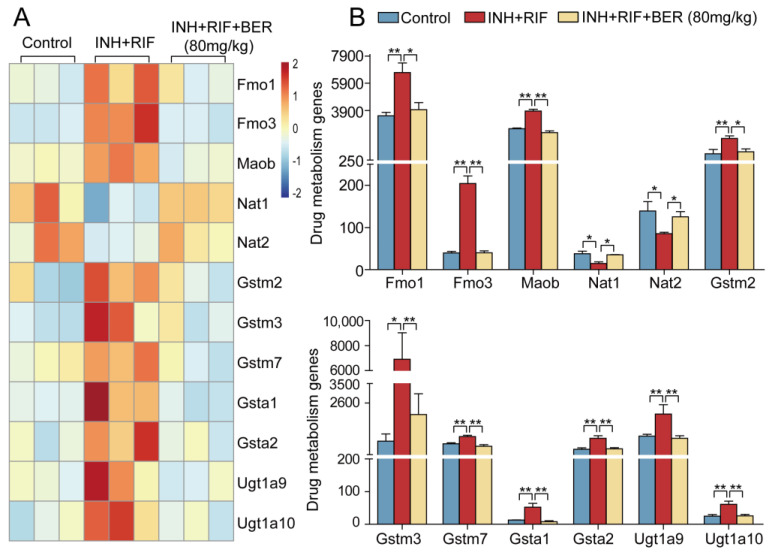
The hierarchical cluster analysis of differentially expressed genes in drug metabolism pathway. (**A**) Heat map of drug metabolism pathway. (**B**) Histograms of drug metabolism pathway. All data were presented as the means ± SEM (n = 3; fold change ≥ 1.5 and * *p* < 0.05, ** *p* < 0.01).

Compared with the Control group, the expressions of drug-phase I genes flavin-containing monooxygenase 1 (Fmo1), flavin-containing monooxygenase 2 (Fmo2) and monoamine oxidase b (Maob) were significantly up-regulated in the INH+RIF group. Compared with the INH+RIF group, they were significantly down-regulated by the BER intervention. Compared with the Control group, the expressions of phase II genes N-acetyltransferase 1 (Nat1) and N-acetyltransferase 2 (Nat2) were significantly down-regulated in the INH+RIF group. Compared with the INH+RIF group, they were significantly up-regulated after the BER intervention. Compared with the Control group, the gene expressions of glutathione S-transferase m2 (Gstm2), glutathione S-transferase m3 (Gstm3), glutathione S-transferase m7 (Gstm7), glutathione S-transferase α 1 (Gsta1), glutathione S-transferase α 2 (Gsta2), UDP glucuronosyltransferase family 1 member A9 (Utg1a9) and UDP glucuronosyltransferase family 1 member A10 (Utg1a10) were significantly up-regulated in the INH+RIF group. Compared with the INH+RIF group, they were significantly down-regulated after the BER intervention (Figure 3A,B).

#### 2.2.3. Effects of BER on Bile Acid Metabolism Genes

**Figure 4:**
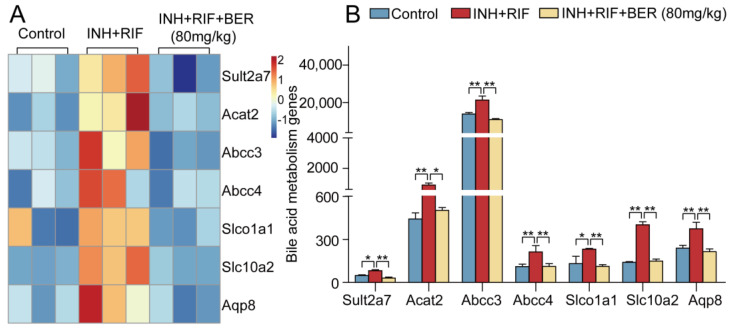
The hierarchical cluster analysis of differentially expressed genes in bile acid metabolism. (**A**) Heat map of bile acid metabolism. (**B**) Histograms of bile acid metabolism. All data were presented as the means ± SEM (n = 3; fold change ≥ 1.5 and * *p* < 0.05, ** *p* < 0.01).

Compared with the Control group, the expressions of the bile acidproduction-related genes sulfotransferase 2a7 (Sult2a7) and acetyl-CoA acetyltransferase 2 (Acat2) were significantly up-regulated in the INH+RIF group. Compared with the INH+RIF group, they were significantly down-regulated after the BER intervention. Compared with the Control group, the expressions of bile acid transport-related genes ATP binding cassette subfamily C member 3 (Abcc3), ATP binding cassette subfamily C member 4 (Abcc4), solute carrier organic anion transporter family 1 member a1 (Slco1a1), solute carrier family 10 member a2 (Slc10a2) and aquaporin 8 (Aqp8) were significantly up-regulated in the INH+RIF group. Compared with the INH+RIF group, they were significantly down-regulated after the BER intervention (Figure 4A,B).

#### 2.2.4. Effects of BER on Nrf2 Pathway Genes

**Figure 5:**
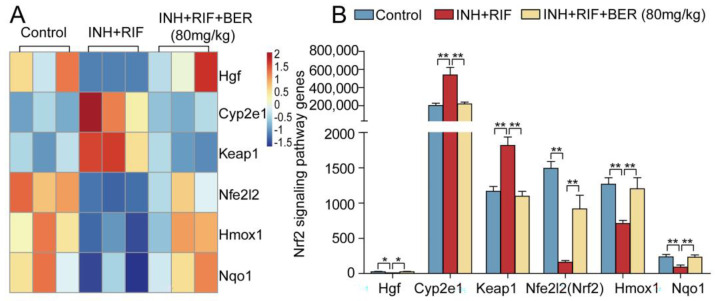
The hierarchical cluster analysis of differentially expressed genes in the Nrf2 pathway. (**A**) Heat map of Nrf2 pathway. (**B**) Histograms of Nrf2 pathway. All data were presented as the means ± SEM (n = 3; fold change ≥ 1.5 and * *p* < 0.05, ** *p* < 0.01).

Compared with the Control group, the gene expressions of cytochrome p450 2e1 (Cyp2e1) and Kelch-like ECH-associated protein-1 (Keap1) were significantly up-regulated, while hepatocyte growth factor (Hgf), nuclear factor erythroid 2-related factor 2 (Nfe2l2, Nrf2), heme oxygenase-1 (Hmox1) and quinone oxidoreductase 1 (Nqo1) were significantly down-regulated in the INH+RIF group. Compared with the INH+RIF group, Cyp2e1 and Keap1 were significantly down-regulated, while Hgf, Nrf2, Hmox1 and Nqo1 were up-regulated after the BER intervention (Figure 5A,B).

#### 2.2.5. Effects of BER on TLR4 Pathway Genes

**Figure 6:**
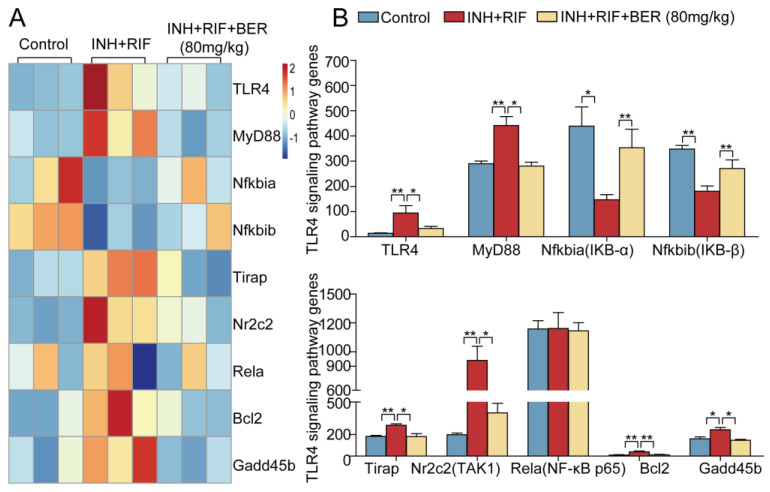
The hierarchical cluster analysis of differentially expressed genes in TLR4 pathway. (**A**) Heat map of TLR4 pathway. (**B**) Histograms of TLR4 pathway. All data were presented as the means ± SEM (n = 3; fold change ≥ 1.5 and * *p* < 0.05, ** *p* < 0.01).

Compared with the Control group, the gene expressions of Toll-like receptor 4 (TLR4), myeloid differentiation primary response 88 (MyD88), toll-interleukin 1 receptor domain-containing adaptor protein (Tirap), nuclear receptor subfamily 2 group C member 2 (Nr2c2, TAK1), B cell leukemia/lymphoma 2 (Bcl2), growth arrest and DNA-damage-inducible 45 β (Gadd45b) were significantly up-regulated, while human nuclear factor κB inhibitor protein α (Nfkbia, IKB-α) and human nuclear factor κB inhibitor protein β (Nfkbib, IKB-β) were significantly down-regulated in the INH+RIF group. Compared with the INH+RIF group, TLR4, MyD88, Tirap, Nr2c2 (TAK1), Bcl2 and Gadd45b were significantly down-regulated, while Nfkbia (IKB-α) and Nfkbib (IKB-β) were significantly up-regulated after the BER intervention. There was no significant difference in the expression of nuclear factor-κB p65 (NF-κB p65, Rela) among all groups (Figure 6A,B).

### 2.3. Verification Experiment

#### 2.3.1. The mRNA Expressions in Key Pathways Were Verified by qRT-PCR

**Figure 7:**
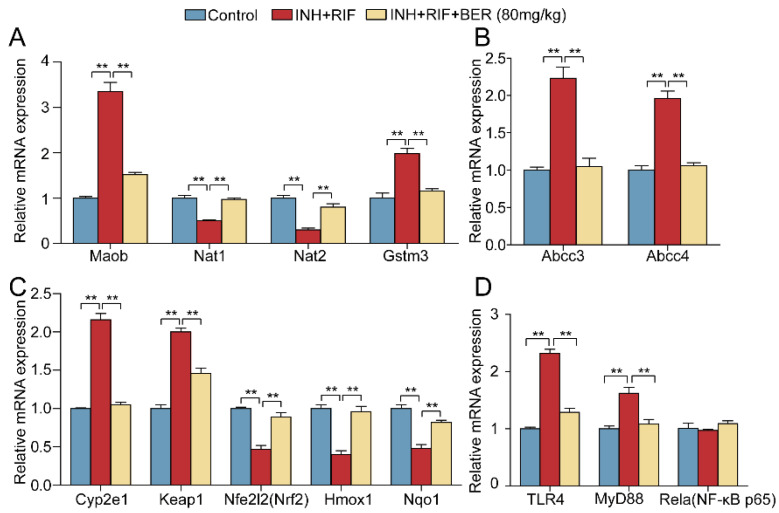
Hepatic mRNA expressions of selected differentially expressed genes. (**A**) Relative mRNA expressions of Maob, Nat1, Nat2 and Gstm3. (**B**) Relative mRNA expressions of Abcc3 and Abcc4. (**C**) Relative mRNA expressions of Cyp2e1, Keap1, Nfe2l2 (Nrf2), Hmox1 and Nqo1. (**D**) Relative mRNA expressions of TLR4, Myd88 and Rela (NF-κB p65). The relative expressions of mRNA were normalized against the Control group, with GAPDH as the internal reference. All data were presented as the mean ± SEM (n = 3; ** *p* < 0.01).

To validate the results of RNA sequencing, qRT-PCR was used to detect the mRNA expressions of several important genes in the above pathway. Compared with the Control group, mRNA expressions of drug-metabolizing-related genes Maob and Gstm3 were significantly up-regulated in the INH+RIF group (increased by 235% and 96%, respectively). Compared with the INH+RIF group, they were significantly down-regulated after the BER intervention (reduced by 54.6% and 41.4%, respectively) (Figure 7A). Compared with the Control group, mRNA expressions of Nat1 and Nat2 were down-regulated in the INH+RIF group (reduced by 50% and 30%, respectively). Compared with the INH+RIF group, they were significantly up-regulated after the BER intervention (increased by 94% and 166%, respectively) (Figure 7A). The mRNA expressions of bile acid metabolizing-related genes Abcc3 and Abcc4 were significantly up-regulated in the INH+RIF group (increased by 123% and 96%, respectively). Compared with the INH+RIF group, they were significantly down-regulated after the BER intervention (reduced by 52.9% and 45.9%, respectively) (Figure 7B). Compared with the Control group, mRNA expressions of Nrf2 pathway-related genes Cyp2e1 and Keap1 were significantly up-regulated in the INH+RIF group (increased by 116% and 100%, respectively). Compared with the INH+RIF group, they were significantly down-regulated after the BER intervention (were reduced by 51.4% and 27%, respectively). The mRNA expressions of Nfe2l2 (Nrf2), Hmox1 (HO-1) and Nqo1 were significantly down-regulated in the INH+RIF group (reduced by 53%, 60% and 52%, respectively). Compared with the INH+RIF group, they were significantly up-regulated after the BER intervention (increased by 89.4% and 140% and 70.8%, respectively). (Figure 7C). Compared with the Control group, the mRNA expressions of TLR4 and Myd88 were significantly up-regulated (increased by 132% and 62%, respectively). Compared with the INH+RIF group, they were significantly down-regulated after the BER intervention (reduced by 44.4% and 33.3%, respectively). (Figure 7D). There was no significant difference in mRNA expression of Rela (NF-κB p65) (Figure 7D). The above results were consistent with those of the RNA sequencing.

#### 2.3.2. The Protein Expressions of the Nrf2 Pathway Were Verified by Western Blot

**Figure 8:**
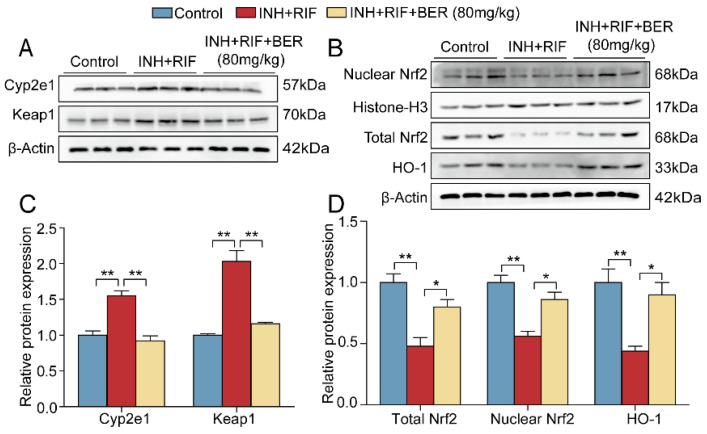
Effect of BER on Nrf2 pathway in liver tissue. (**A**–**D**) Relative protein expressions of Cyp2e1, Keap1, Total Nrf2, Nuclear Nrf2 and HO-1 in liver tissue. The relative protein expressions were normalized against the Control group, with β-Actin as the internal reference. All data were presented as the mean ± SEM (n = 3; * *p* < 0.05, ** *p* < 0.01).

Compared with the Control group, the protein expressions of Cyp2e1 and Keap1 were significantly up-regulated (increased by 55% and 100.3%, respectively), and the protein expressions of nuclear Nrf2, total Nrf2 and HO-1 were significantly down-regulated in the INH+RIF group (reduced by 44% and 50% and 56%, respectively). Compared with the INH+RIF group, the protein expressions of Cyp2e1 and Keap1 were significantly down-regulated (reduced by 40.6% and 42.8%, respectively), while the protein expressions of nuclear Nrf2, total Nrf2 and HO-1 were significantly up-regulated after the BER intervention (increased by 53.6%, 66% and 104.5%, respectively) (Figure 8A–D). The above results were consistent with those of the RNA sequencing.

#### 2.3.3. The Protein Expressions of the TLR4 Pathway Were Verified by Western Blot

**Figure 9:**
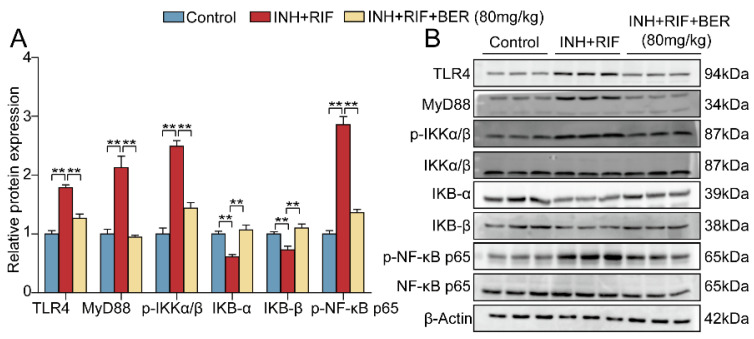
Effect of BER on TLR4 pathway in liver tissue. (**A**,**B**) Relative protein expressions of TLR4, MyD88, p-IKKα/β, IKKα/β, IKB-α, IKB-β, p-NF-κB p65, and NF-κB p65 in liver tissue. The relative protein expressions were normalized against the Control group, with β-Actin as the internal reference. All data were presented as the mean ± SEM (n = 3; ** *p* < 0.01).

Compared with the Control group, the protein expressions of TLR4, MyD88, phosphorylated IκB kinase α/β (p-IKKα/β), and phosphorylated nuclear factor-κB p65 (p-NF-κB p65) were significantly up-regulated (increased by 79%, 113%, 153% and 83%, respectively) and IKB-α, IKB-β were significantly down-regulated in the INH+RIF group (reduced by 39% and 27%, respectively). Compared with the INH+RIF group, TLR4, MyD88 and p-IKKα/β were significantly down-regulated (reduced by 29%, 55.4%, 56.1% and 43.2%, respectively), while IKB-α and IKB-β were significantly up-regulated after the BER intervention (increased by 75.4% and 50.7%, respectively) (Figure 9A,B). The above results were consistent with those of RNA sequencing.

## 3. Discussion

INH and RIF are considered to be potentially hepatotoxic and can induce liver damage. A perennial application of INH and RIF can cause hepatocellular damage and cholestasis [18]. In this study, the INH+RIF-induced liver injury was constructed according to other studies [19]. After the BER intervention, the pathological changes of mouse liver tissue were significantly improved. In addition, RNA sequencing was performed to investigate the mechanism of action. The results of RNA sequencing showed that the mechanism of BER alleviating INH+RIF-induced liver injury was related to the regulation of drug-metabolizing enzymes, cholestasis, oxidative stress and inflammation. Furthermore, we verified the results of RNA sequencing by qRT-PCR and Western blot, and further elucidated the mechanism of liver protection of BER in terms of Nrf2 pathway and TLR4 pathway.

HE staining showed that INH+RIF induced focal necrosis and inflammatory infiltration in mouse liver tissue, indicating successful model construction (Figure 1F). When cell-membrane permeability is increased, ALT and AST from the hepatocytes are released into the blood, resulting in increased serum activity [20]. Therefore, ALT and AST are important and sensitive biochemical indicators in liver function. In this study, ALT and AST activities were significantly down-regulated after the BER intervention (Figure 1A). This confirmed the protective effects of BER on INH+RIF-induced liver injury. Clinically, cholestasis is the most common type of liver injury caused by antituberculosis drugs. Bilirubin excretion was competitively inhibited by INH+RIF, with elevated AKP, TBA, DBIL and TBIL. In this study, serum levels of AKP, TBA, DBIL and TBIL were decreased by BER (Figure 1B). It suggested that BER alleviated INH+RIF-induced liver injury by ameliorating cholestasis.

It has been shown that INH+RIF-induced liver injury was closely related to abnormal drug metabolism, cholestasis, oxidative stress and inflammatory response [18]. Subsequently, RNA sequencing was processed in liver tissue to obtain the differentially expressed genes. They were focused on the effects of BER on the drug metabolism pathway, bile acid metabolism pathway, Nrf2 pathway and TLR4 pathway.

The drug metabolism process can be divided into phase I and phase II. Phase I includes oxidation, reduction and hydrolysis. Phase II includes glucuronidation, sulphation and glutathione coupling. Drug metabolism genes may be the main targets of INH+RIF-induced liver injury. In this study, some genes related to drug metabolism were changed in liver tissues after INH+RIF induction. N-acetyltransferases play an important role in the metabolism of INH. INH is metabolized by N-acetyltransferases (Nat1, Nat2) to acetyl hydrazine and finally hydrolyzed to non-toxic diacetyl hydrazine for excretion. The induction of INH+RIF leads to the down-regulation of Nat1 and Nat2 expression, which leads to reduced acetylation of INH and production of toxic hydrazine [21,22], which was also observed in this study and reversed by the BER intervention (Figure 3A,B and Figure 7A). Furthermore, due to the overlapping substrate specificity and the unresolved functional significance of the Nat1 and Nat2 allelic variants, caution must be exercised in interpreting the tissue localization of them based on metabolic activity. Numerous studies have examined Nat expression in tissues using RT-PCR [23,24,25], which is similar to our approaches. In this study, the mRNA expression of Nat1 and Nat2 was down-regulated by INH+RIF. This suggests the accumulation of toxic metabolites in the liver, which led to liver damage. Meanwhile, INH was further oxidized by Cyp2e1 and excessive acetyl radicals were generated. RIF provides acetyl groups for INH and synergically induced liver injury. Flavin-containing monooxygenase (Fmo1, Fmo3) is a kind of enzyme similar to CYPs, and it can catalyze the oxidation of N, P, S (e.g., GSH) substances. In addition, Maob is also a very important drug oxidase. In this study, the Fmo1, Fmo3 and Maob were abnormally expressed in INH+RIF-induced liver injury and BER changed them (Figure 3A,B and Figure 7A). Glutathione S-transferases (GSTs), highly expressed detoxifying enzymes in the liver, catalyze glutathione to bind to electrophiles (such as ROS and free radicals), which ultimately maintain redox equilibrium. In this study, it was found that the expressions of Gstm2, Gstm3, Gstm7, Gsta1 and Gsta2 were up-regulated by INH+RIF, which might be a stress response for cells to get rid of toxic substances (Figure 3A,B). However, the BER intervention reduced the expression of GSTs, and we hypothesized that BER inhibited the production of toxic substances, leading to a relative decline in GST expression. UDP glucuronosyltransferase-family genes (UGTs) are essential for the metabolism and clearance of many endogenous and exogenous compounds, including bile acid, bilirubin, fatty acid, carcinogens and therapeutic drugs [26]. In this study, Ugt1a9 and Ugtla10 genes were abnormally up-regulated in the INH+RIF group, and they were markedly down-regulated after the BER intervention (Figure 3A,B). We speculated that UGTs might be up-regulated by the body for the sake of adapting to abnormally high levels of bilirubin and bile acid. In other words, BER reduced the levels of bilirubin and bile acid, thus down-regulating the expression of these genes. Moreover, we paid attention to the expression of genes related to the bile acid metabolism pathway.

Bile acid is the main product of cholesterol metabolism. Sulfotransferase (SULTs) and Acat2 are important enzymes that promote cholesterol metabolism and bile acid production. Cholesterol is catalyzed into bile acid by SULTs-mediated sulfosylation. Cholesterol is catalyzed into cholesterol esters by Acat2, which speeds up the production of bile acid. In this study, the expressions of Sult2a7 and Acat2 were abnormally increased after INH+RIF induction, and significantly down-regulated after the BER intervention (Figure 4A,B). Abcc3 and Abcc4, members of the ATP binding box family (ABC), play an important role in bile acid efflux. Their expressions are abnormally increased in certain liver disease states, such as cholestasis, primary biliary cirrhosis and non-alcoholic liver disease [27,28,29]. Therefore, Abcc3 and Abcc4 are considered bile acid effector transporters in the adaptive response to cholestatic injury. The synergistic action of ABC and solute carrier transporter (SLC) are required to maintain bile acid and bilirubin homeostasis. The increased uptake of hepatotoxic drugs may be the result of increased SLC expression [30]. Aqp8, a transmembrane channel protein, restricts the rate of water secretion in the ependymal membrane during biliary secretion. In this study, after induction of INH+RIF, the expressions of genes related to bile acid transport (Abcc3, Abcc4, Slco1a, Slc1oa2, Aqp8) were abnormally up-regulated. These results indicated that the manifestation of INH+RIF-induced liver injury was cholestasis, which was consistent with other studies [31,32]. After the BER intervention, cholestasis in the liver was improved (Figure 4A,B and Figure 7B). It was consistent with the results of this research (Figure 1B). In short, the effect of BER on cholestasis in INH+RIF-induced liver injury was related to the regulation of bile acid synthesis and transport.

Hgf is a substance that can stimulate hepatocyte proliferation and regulate the Nrf2 pathway. Nrf2 is a major transcription factor in response to oxidative stress. Nrf2 affects the expression of nearly 500 genes, which regulate the redox balance factors, detoxifying enzymes, stress response proteins and metabolic enzymes [33]. Once the cell is stimulated, Nrf2 will separate from Keap1 and enter the nucleus. Then, Nrf2 combines with antioxidant elements (ARE) to promote the synthesis of peroxidase enzymes (SOD, GSH-Px). Cyp2e1 is induced by INH+RIF, which increases the release of ROS and weakens the activities of SOD and GSH-Px, thus promoting the production of lipid peroxidation products (MDA). In this study, BER enhanced the activities of SOD and GSH-Px, and reduced the contents of MDA (Figure 1C,D). In addition, BER significantly up-regulated the expressions of Hgf and Nrf2, and markedly down-regulated the expressions of Cyp2e1 and Keap1 (Figure 5A,B, Figure 7C and Figure 8A–D). It suggested that BER played an antioxidant role. Nqo1 and HO-1, as downstream factors of Nrf2, also play an important antioxidant role. In this study, BER promoted the generation of HO-1 and Nqo1 (Figure 5A,B, Figure 7C and Figure 8B,D). This suggests that BER increased the activity of peroxidase and reduced lipid peroxidation by regulating the Nrf2 pathway, which ultimately alleviated liver cell death.

Oxidative stress and inflammation are often closely related. The TLR4 signaling pathway is a classic pathway associated with inflammation. It has been reported that inflammation and apoptosis were alleviated by inhibiting the TLR4/NF-κB pathway, which ameliorated acute liver injury caused by lipopolysaccharides [34]. Therefore, in this study, it was explored the role of the TLR4 pathway in liver injury induced by INH+RIF. TLR4 induces macrophages to differentiate into M1 phenotypes, which then produces inflammatory cytokines [35]. In this research, the expressions of TLR4, TNF-α, IL-6 and IL-1β in the liver tissue of mice treated with INH+RIF were abnormally increased. After BER treatment, their expressions were significantly down-regulated (Figure 1E, Figure 6A,B, Figure 7D and Figure 9A,B). There is a close relationship between TLR4 and NF-κB p65. Upon receiving the stimulus signal, TLR4 conducts the signal through a MyD88-dependent pathway [36]. MyD88 then interacts with Tirap to trigger the autophosphorylation of IRAK1 and IRAK4, followed by the activation of TAK1. Next, IKK is phosphorylated, leading to the inhibition of IKB-α/β. Gradually, the NF-κB p65 subunit is dissociated into the nucleus and united to target genes. Finally, it promotes the secretion of inflammatory cytokines [37]. In this study, gene or protein expressions of MyD88 and p-IKKα/β were abnormally increased, and IKB-α and IKB-β were inhibited after induction by INH+RIF, while the BER intervention significantly inhibited MyD88 and p-IKKα/β and increased IKB-α and IKB-β (Figure 6A,B and Figure 9A,B). In short, BER alleviated INH+RIF-induced inflammatory response in liver tissue by inhibiting the TLR4 pathway.

RNA sequencing helped us fully understand the protective effect of BER on INH+RIF-induced liver injury. In general, abnormal drug metabolism, cholestasis, oxidative stress and inflammatory responses were important triggers of INH+RIF-induced liver injury. BER was an active compound with antioxidant and anti-inflammatory effects in this study.

## 4. Materials and Methods

### 4.1. Animal Experiments

The male mice (6–8 weeks, 18–20 g) used in this study were purchased from Hunan SJA Laboratory Animal Co. All mice were placed at a temperature of 22–25 °C and relative humidity of 50–60%, and guaranteed 12 h of alternating light-darkness.

**Figure 10:**
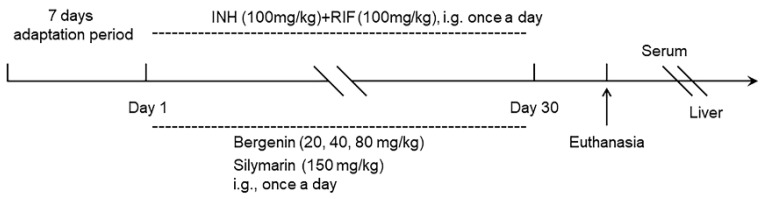
An overview of animal experiments.

The 70 mice were randomly divided into 7 groups of 10 mice each group, including the Control group, INH+RIF group, INH+RIF+Silymarin group, INH+RIF+BER (20 mg/kg) group, INH+RIF+BER (40 mg/kg) group, INH+RIF+BER (80 mg/kg) group and BER (80 mg/kg) group. Except for the Control group and BER (80 mg/kg) group, mice were given 100 mg/kg INH (Purity: HPLC ≥ 98%; 54-85-3, Aladdin Biochemical Technology, Co. Ltd., Shanghai, China) and 100 mg/kg RIF (Purity: HPLC ≥ 97%, 13292-46-1, Aladdin Biochemical Technology Co., Ltd., Shanghai, China), followed by the corresponding dose of BER (Purity: HPLC ≥ 98%; BD5122; Bidepharm, Shanghai, China) and Silymarin (150 mg/kg, Purity: UV ≥ 80%; MB5982; Meilunbio, Dalian, China) for 30 days. Mice were given 0.5% sodium carboxymethylcellulose solution (10 mL/kg) in the Control group. The BER (80 mg/kg) group was given 80 mg/kg BER for 30 days. All drugs were suspended in 0.5% sodium carboxymethyl cellulose solution. After 30 days, the blood and tissues were collected for subsequent experiments. The blood samples were centrifuged at 4500 rpm for 15 min and serums were collected for biochemical analysis. Some liver tissues were quickly immersed in liquid nitrogen for RNA sequencing, qRT-PCR and Western blot experiments. In addition, some tissues of the liver, brain, heart, spleen, lung and kidney were fixed in 4% paraformaldehyde solution for histological analysis. An overview of animal experiments was shown in Figure 10.

### 4.2. Liver Histological Analysis

The liver tissues of mice were fixed with 4% paraformaldehyde for 48 h. After gradient ethanol dehydration and paraffin embedding, the wax blocks were cut into slices with a thickness of 4 μm. HE staining was performed and the pathological changes of the liver tissues were observed under a BX51 light microscope (Olympus; Tokyo, Japan).

### 4.3. Analysis of Serum Samples and Liver Tissue Samples

According to the instructions, the serums levels of alanine aminotransferase (ALT, C009-2-1), aspartate aminotransferase (AST, C010-2-1), alkaline phosphatase (AKP, A059-2-2), bile acid (TBA, E003-2-1), direct bilirubin (DBIL, C019-2-1) and total bilirubin (TBIL, C019-1-1) were determined. The activities or contents of superoxide dismutase (SOD, A001-3-2), glutathione peroxidase (GSH-Px, A005-1-2) and malondialdehyde (MDA, A003-1-2) in the liver were also measured (Nanjing Jiancheng Bioengineering Research Institute, Nanjing, China). The contents of TNF-α (MM-0132M1), (MM-0132M1), IL-6 (MM-0163M1) and IL-1β (MM-0040M1) in liver tissues were determined by enzyme-linked immunosorbent assay (Meimian Industrial Co., Ltd., Wuhan, China).

### 4.4. RNA Sequencing Analysis

Liver tissue samples (the Control group, INH+RIF group and INH+RIF+BER (80 mg/kg) group, with 3 samples in each group) were sequenced by PanoMIX (Suzhou, China). A 300 bp fragment was obtained by Oligo (dT) magnetic bead enrichment and ion interruption. The first strand of cDNA (as a template for the second strand of cDNA) was synthesized using a 6-base random primer and reverse transcriptase. After library construction, library fragments were enriched using PCR amplification, followed by library selection based on the fragment size, with the library size at 450 bp. Next, libraries were checked for quality by Agilent 2100 Bioanalyzer, and then total library concentration and effective library concentration were tested. The mixed libraries were uniformly diluted to 2 nM and formed into single-stranded libraries by base denaturation. Finally, the libraries were sequenced by double-end (Paired-end, PE) sequencing based on the Illumina sequencing platform. Tophat2 was used to compare clean reads with the reference genome. Then, DESeq (v1.38.3) software was used for differential expression analysis based on the read count of genes. *p* ≤ 0.05 and foldchange ≥ 1.5 were set as the thresholds.

### 4.5. Quantitative Real-Time Polymerase Chain Reaction (qRT-PCR)

The total RNA of liver tissues was reverse transcribed into cDNA on the basis of the HiFiScript gDNA Removal cDNA Synthesis Kit (Beijing ComWin Biotech Co., Ltd., Beijing, China). According to the reaction conditions of the SYBR Green quantitative PCR kit (Beijing ComWin Biotech Co., Beijing, China), the amplified cDNA was detected by Quant Studio3 real-time quantitative PCR instrument (Thermo Fisher Scientific, Waltham, MA, USA). The relative expression of each gene was calculated using GAPDH as the internal reference (formula: 2^−ΔΔCt^). The primer sequences are shown in Table 1 (The Beijing Genomics Institute, Shenzhen, China).

### 4.6. Western Blot

A mixture of 60 mg of liver tissues mixed with 1 mL of RIPA lysis solution was homogenized using a biological sample homogenizer (Shanghai Jingxin Industrial Development Co., Shanghai, China). The supernatant was obtained by centrifugation at 12,000 rpm for 30 min. Then, the protein concentration was determined according to the instructions of the BCA kit. The proteins were separated by polyacrylamide gel electrophoresis (SDS-PAGE) and then transferred to the NC membranes. Next, the NC membranes were blocked in 5% skim milk powder solution for 2 h and incubated overnight with primary antibodies: MyD88 (bs-1047R); IKB-β (bs-10246R); p-IKK-α/β (bs-3237R); p-NF-κB p65 (bsm-52178R) (Bioss, Beijing, Chain); NF-κB p65 (sc-8008, Santa Cruz, CA, USA); TLR-4 (19811-1-AP); IKB-α (18220-1-1AP); Nrf2 (16396-1-AP); HO-1 (10701-1-AP); Cyp2e1 (19937-1-AP) (Proteintech, Wuhan, China); Histon-H3 (ab1791); Keap1 (ab227828); IKK-α/β (ab178870) (Abcam, Cambridge, UK); and β-Actin (TA-09, Nakasugi Golden Bridge, Beijing, China). The next day, the NC membranes were incubated with the secondary antibodies (Goat Anti-Mouse IgG ZB-2305 or Goat Anti-Rabbit IgG ZB-2301, Sam Golden, Beijing, China) for 60 min. Finally, the NC membranes were imaged in a GeneGnome XRQ professional chemiluminescence imaging system (Syngene, Cambridge, UK). The protein expressions were quantified using Image-J and normalized with β-Actin as an internal reference.

### 4.7. Statistical Analysis

IBM SPSS 20.0 software was used for statistical analysis. Unless otherwise stated, all data were expressed as mean ± standard error of mean (SEM). Normal distributions were assessed using the Shapiro–Wilk test. For two-group comparisons with equal variance as determined by the F-test, an unpaired two-tailed t-test was used. Non-normally distributed data were assessed by Mann–Whitney U test. For comparisons between multiple groups, normally distributed and equal variance data were analyzed by one-way analysis of variance (ANOVA). Non-normally distributed or unequal variance data were analyzed by Kruskal–Wallis univariate analysis. *p* < 0.05 was considered statistically significant.

## 5. Conclusions

**Figure 11:**
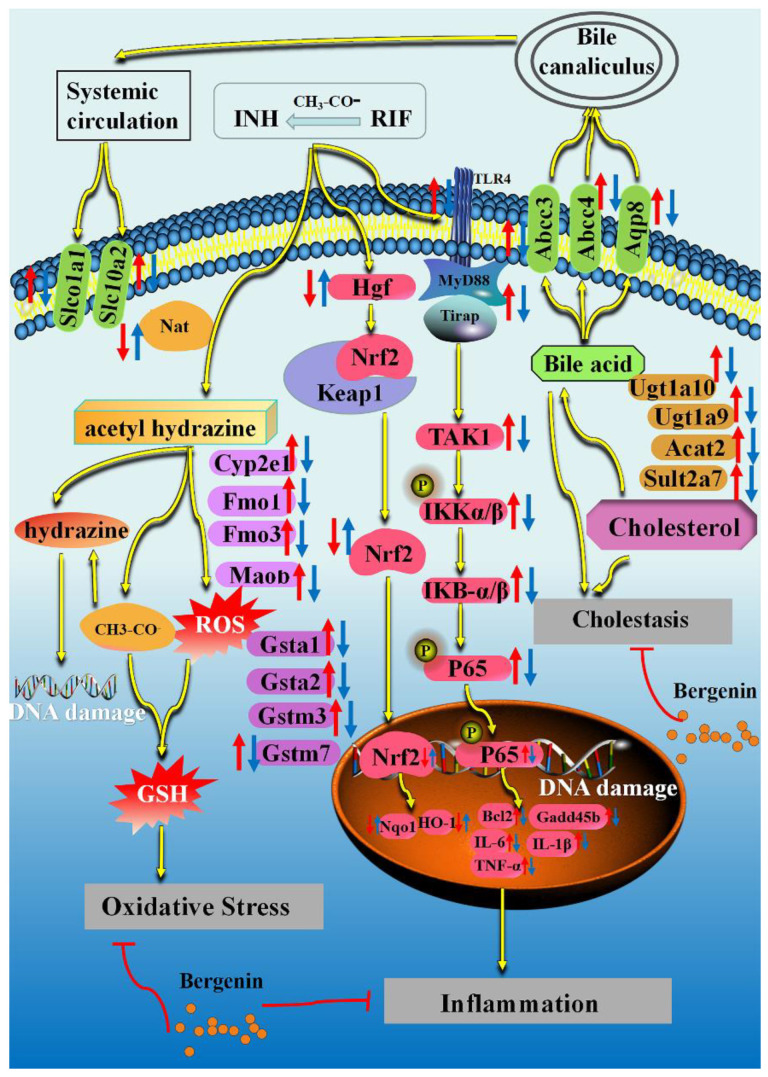
Proposed model depicting the pathogenesis of INH+RIF-induced liver injury in mice, and the underlying mechanisms of BER in improving liver injury. However, there was no data in this study to support the upstream and downstream relationship of each factor, which was inferred according to the references. The Control group vs. the INH+RIF group: ↑ up-regulated; ↓ down-regulated. The INH+RIF group vs. the INH+RIF+BER (80 mg/kg) group: ↑ up-regulated; ↓ down-regulated.

In this study, the molecular mechanism of BER alleviating INH+RIF-induced liver injury was revealed. BER improved cholestasis, oxidative stress and inflammation, which was related to the regulation of drug-metabolizing enzymes, bile acid metabolism, the Nrf2 pathway and the TLR4 pathway (Figure 11). This provides new methods for the clinical treatment of INH+RIF-induced liver injury.

## Figures and Tables

**Table 1:** Primer sequences.

Gene	Primer	Sequence (5′-3′)
Maob	Forward	ATGAGCAACAAAAGCGATGTGA
	Reverse	TCCTAATTGTGTAAGTCCTGCCT
Nat1	Forward	AGATGCGAGCAGTTCCTTTTG
	Reverse	CCTGTACTAGAAGGTGGACCATT
Nat2	Forward	ACACTCCAGCCAATAAGTACAGC
	Reverse	GGTAGGAACGTCCAAACCCA
Gstm3	Forward	CCCCAACTTTGACCGAAGC
	Reverse	GGTGTCCATAACTTGGTTCTCCA
Abcc3	Forward	CTGGGTCCCCTGCATCTAC
	Reverse	GCCGTCTTGAGCCTGGATAAC
Abcc4	Forward	AGGAGCTTCAACGGTACTGG
	Reverse	GCCTTTGTTAAGGAGGGCTTC
Cyp2e1	Forward	CGTTGCCTTGCTTGTCTGGA
	Reverse	AAGAAAGGAATTGGGAAAGGTCC
Keap1	Forward	TGCCCCTGTGGTCAAAGTG
	Reverse	GGTTCGGTTACCGTCCTGC
Nrf2	Forward	TCTTGGAGTAAGTCGAGAAGTGT
	Reverse	GTTGAAACTGAGCGAAAAAGGC
Hmox1	Forward	AAGCCGAGAATGCTGAGTTCA
	Reverse	GCCGTGTAGATATGGTACAAGGA
Nqo1	Forward	AGGATGGGAGGTACTCGAATC
	Reverse	AGGCGTCCTTCCTTATATGCTA
TLR4	Forward	ATGGCATGGCTTACACCACC
	Reverse	GAGGCCAATTTTGTCTCCACA
MyD88	Forward	TCATGTTCTCCATACCCTTGGT
	Reverse	AAACTGCGAGTGGGGTCAG
NF-κB p65	Forward	AGGCTTCTGGGCCTTATGTG
	Reverse	TGCTTCTCTCGCCAGGAATAC

## Data Availability

Data are contained within the article.
